# SVIP Induces Localization of p97/VCP to the Plasma and Lysosomal Membranes and Regulates Autophagy

**DOI:** 10.1371/journal.pone.0024478

**Published:** 2011-08-31

**Authors:** Yang Wang, Petek Ballar, Yongwang Zhong, Xuebao Zhang, Chao Liu, Ying-Jiu Zhang, Mervyn J. Monteiro, Jun Li, Shengyun Fang

**Affiliations:** 1 Key Laboratory for Molecular Enzymology and Engineering of Ministry of Education, Jilin University, Changchun, People's Republic of China; 2 Center for Biomedical Engineering and Technology, University of Maryland, Baltimore, Maryland, United States of America; 3 Department of Physiology, University of Maryland, Baltimore, Maryland, United States of America; 4 Department of Neurology, Vanderbilt University, Nashville, Tennessee, United States of America; Johns Hopkins School of Medicine, United States of America

## Abstract

The small p97/VCP-interacting protein (SVIP) functions as an inhibitor of the endoplasmic reticulum (ER)-associated degradation (ERAD) pathway. Here we show that overexpression of SVIP in HeLa cells leads to localization of p97/VCP at the plasma membrane, intracellular foci and juxtanuclear vacuoles. The p97/VCP-positive vacuolar structures colocalized or associated with LC3 and lamp1, suggesting that SVIP may regulate autophagy. In support of this possibility, knockdown of SVIP diminished, whereas overexpression of SVIP enhanced LC3 lipidation. Surprisingly, knockdown of SVIP reduced the levels of p62 protein at least partially through downregulation of its mRNA, which was accompanied by a decrease in starvation-induced formation of p62 bodies. Overexpression of SVIP, on the other hand, increased the levels of p62 protein and enhanced starvation-activated autophagy as well as promoted sequestration of polyubiquitinated proteins and p62 in autophagosomes. These results suggest that SVIP plays a regulatory role in p97 subcellular localization and is a novel regulator of autophagy.

## Introduction

p97/VCP is a homohexameric AAA ATPase that is involved in a myriad of cellular functions [1]–[3]. The specificity of p97/VCP function is regulated by differential interaction with its adaptor proteins [4]. The small p97/VCP-interacting protein (SVIP) is one such adaptor [5]. Earlier studies showed that SVIP inhibited the function of p97/VCP in ER-associated degradation (ERAD), the pathway by which misfolded proteins are removed from the ER via proteasomal degradation [6].

p97/VCP plays a central role in ERAD. p97/VCP along with its adaptor Npl4 or Ufd1/Npl4 heterodimer bind and extract polyubiquitinated proteins from the ER for degradation by the cytosolic proteasomes [7]–[9]. To conduct this function, p97/VCP has to be recruited to the ER from the cytosol [7]–[9]. The ubiquitin ligases, gp78 and Hrd1, are involved in this recruitment as they both contain p97/VCP-binding motifs [10], [11]. Hrd1 contains a p97/VCP-binding motif (VBM), whereas gp78 contains a p97/VCP-interacting motif (VIM) [4], [11]. Binding to these motifs would position p97/VCP ideally for extraction of the ER substrate following its ubiquitination by the ubiquitin ligases. We previously showed that SVIP also contains a VIM and that it competes with gp78 for binding to p97/VCP, thereby inhibiting ERAD [6]. The ERAD-inhibitory role of SVIP is apparently reduced through downregulation of the SVIP protein upon induction of ER stress [6]. Paradoxically, prolonged ER stress, a condition associated with accumulation of misfolded proteins in the ER, significantly upregulates SVIP, which is expected to severely inhibit ERAD [6]. The present study suggests that this upregulation may promote autophagy.

Autophagy is a highly conserved quality control system in eukaryotes for non-selective removal of long-lived proteins, proteins aggregates and damaged organelles [12]–[14]. In response to an initiation signal, such as starvation and oxidative stress, a double membrane structure called isolation membrane or phagophore is formed [12]–[14]. The phagophore then expands and eventually seals leading to the formation of a double membrane-bound vesicle known as autophagosome, which completes sequestering cargos. The autophagosome fuses with lysosomes forming autolysosomes where the cargos are degraded. p97/VCP facilitates autophagosome-lysosome fusion [15], [16]. The autophagic degradation process is essential for maintaining cellular homeostasis and for orchestration of cellular responses to stress [12]–[14]. Autophagy is regulated by a set of proteins known as Atg (autophagy-related genes) proteins. A subset of the Atg proteins forms the core machinery that drives the initiation, expansion and closure of phagophore to form the autophagosome. Among Atg proteins, pro-Atg8/LC3, is processed to LC3II that is conjugated to phosphatidylethanolamine in the phagophore membrane by a reaction that requires the E1-like enzyme Atg7, the E2-like enzyme Atg3 and the E3-like enzyme Atg16L. LC3II is attached to both the inner and outer membranes of autophagosomes and the inner membrane-attached LC3II is degraded along with cargos. Therefore, LC3II is a widely used marker for autophagosomes and changes in LC3II levels is commonly used for monitoring the flow of autophagic degradation [12]–[14]. In addition, autophagy is also involved in degradation of ubiquitinated proteins. In this case, two ubiquitin-binding proteins, p62/sequestosome and NBR1 sequester ubiquitinated proteins into autophagosomes [17], [18]. As for LC3II, both p62 and NBR1 are degraded along with their cargo proteins.

In this study, we found that overexpression of SVIP markedly relocalizes p97/VCP, lamp-1, LC3, p62 and polyubiquitin in cells. By knockdown and overexpression of SVIP, we identify SVIP as novel regulator of autophagy via modulating LC3 processing, p62 expression and sequestration of polyubiquitinated proteins into autophagosomes.

## Results

### SVIP is highly expressed in central nervous system

**Figure 1:**
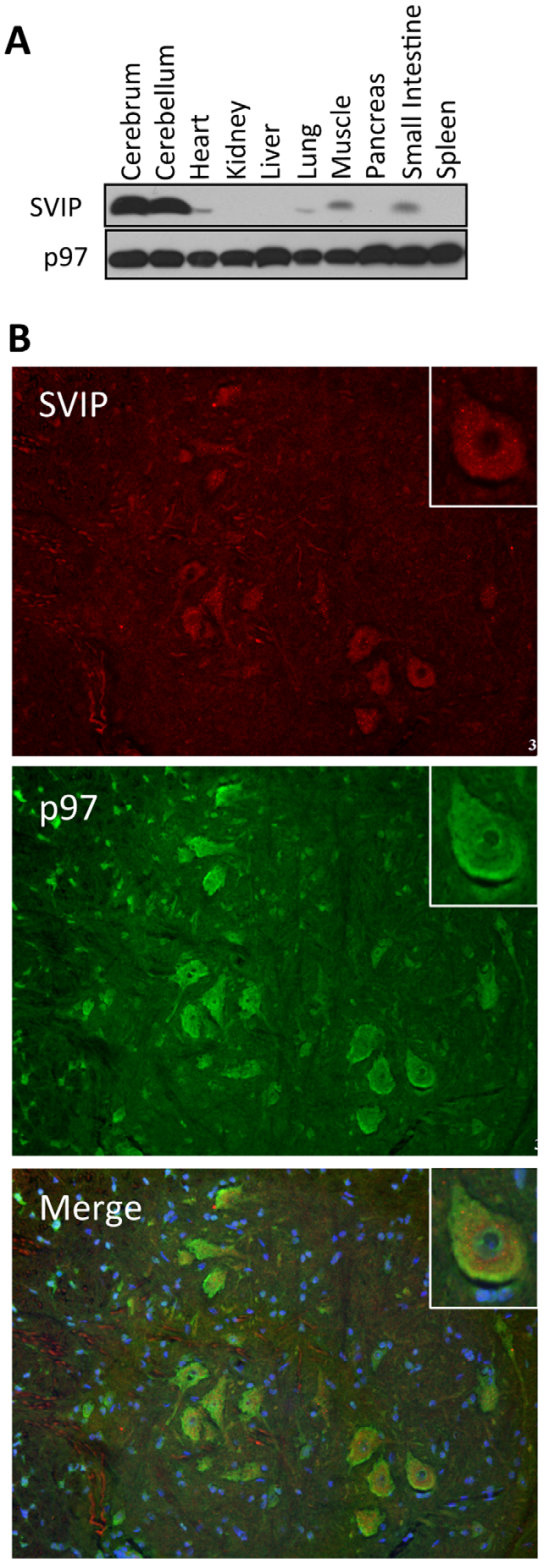
SVIP is highly expressed in brain and colocalizes with p97/VCP in the motor neurons of spinal cord. A. SVIP is highly expressed in brain. Equal amounts of total proteins from each organ extract were analyzed by immunoblotting for SVIP and p97/VCP. B. Colocalization of SVIP and p97/VCP in the motor neurons of spinal cord. Cryostat sections of mouse spinal cord were immunostained for SVIP and p97/VCP. Nuclei were counterstained in blue using DAPI.

To examine the tissue distribution of SVIP, we conducted immunoblotting of tissue extracts made from different mouse organs. We found that SVIP is highly expressed in the cerebrum and cerebellum of the brain. Modest levels were detected in heart, lung, skeletal muscle and small intestine, and undetectable in kidney, liver, pancreas and spleen (Fig. 1A). Immunofluorescence demonstrated that SVIP and p97/VCP colocalize in cytoplasm of the motor neurons in spinal cord. Interestingly, more p97/VCP appeared to localize in the periphery, possibly plasma membranes, of the neurons (Fig. 1B, insets).

### SVIP localizes p97/VCP to the plasma membrane, intracellular foci and vacuoles

**Figure 2:**
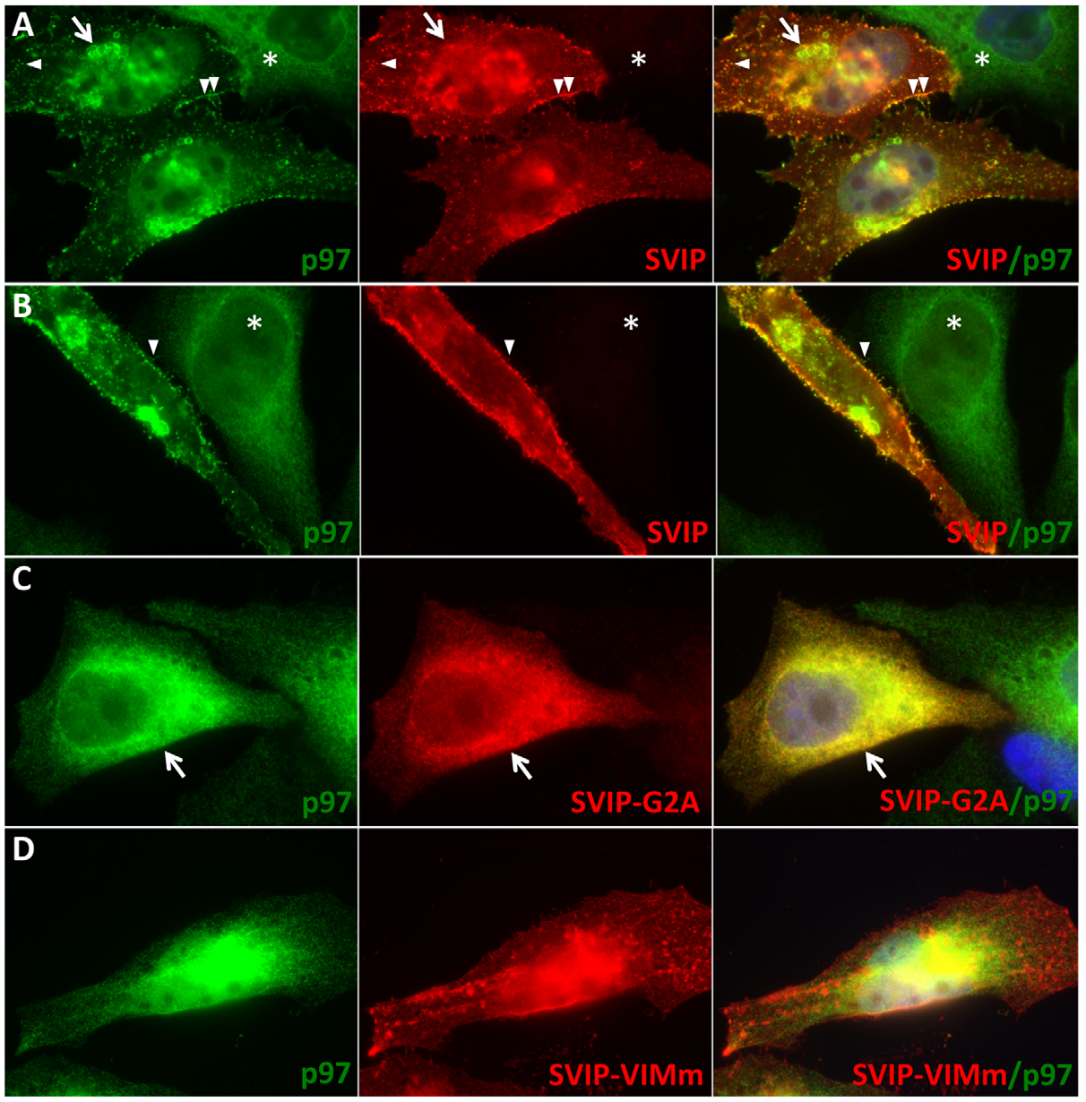
SVIP overexpression relocalizes p97/VCP from cytosol to plasma membrane, cytoplasmic foci and juxtanuclear vacuoles. A, B. SVIP relocalizes p97/VCP from cytosol to plasma membrane, intracellular foci and vacuoles. Images were taken by focusing on juxtanuclear vacuoles (A) or cell surface (B). Arrow: juxtanuclear p97/VCP-positive vacuoles; Arrowhead: intracellular foci positive for p97/VCP; Double arrowheads: plasma membrane localization of p97/VCP; Asterisks: a non-transfected cell. C, D. Mutation of the myristoylation site (G2A) or VIM (VIMm) diminishes the ability of SVIP to relocalize p97/VCP in cells. Arrow: SVIP-G2A-expressing cell. Nuclei were counterstained in blue using DAPI.

We previously showed that SVIP is anchored to the membrane by myristoylation and that it interacts with p97/VCP through its VIM (p97/VCP-interacting motif) [6]. p97/VCP, on the other hand, is a soluble protein. We asked if SVIP interaction could change p97/VCP localization in cells. p97/VCP is normally localized in the cytoplasm and nucleus (Fig. 2A, B, asterisks). This p97/VCP localization can be regulated only when the expression levels of a particular interacting protein is elevated, for example, by overexpression of Derlin1, VIMP and gp78 [11], [19]. Therefore, we overexpressed SVIP in HeLa cells and stained SVIP and p97/VCP by immunofluorescence. SVIP overexpression induced a marked relocalization of p97/VCP to juxtanuclear vacuoles (Fig. 2A, arrow), intracellular foci (Fig. 2A, arrowhead) and plasma membrane (Fig. 2A, double arrowheads) as well as spine-like structure on the cell surface (Fig. 2A, double arrowheads and 2B, arrowhead). Despite the fact that SVIP induces a marked relocalization of p97/VCP, it only partially colocalized with p97/VCP. SVIP colocalized with p97/VCP in some intracellular foci (Fig. 2A, arrowhead) and spine-like structure on the cell surface (Fig. 2A, double arrowheads and 2B, arrowhead), but not in p97/VCP vacuoles (Fig. 2A, arrow) and tip of the spines (Fig. 2B, arrowhead). These results suggest that some SVIP dissociates from p97/VCP during the process of p97/VCP relocalization. SVIP-induced p97/VCP relocalization was dependent on SVIP myristoylation and the VIM of SVIP, since its localization was not changed in cells expressing SVIP with mutation of either its myristoylation site (G2A) or VIM (VIMm) (Fig. 2C, D). These results suggest that SVIP interaction relocalizes p97/VCP to plasma membrane, membrane-bound vesicles and vacuoles in cells.

### SVIP induces localization of lamp1 to juxtanuclear regions

**Figure 3:**
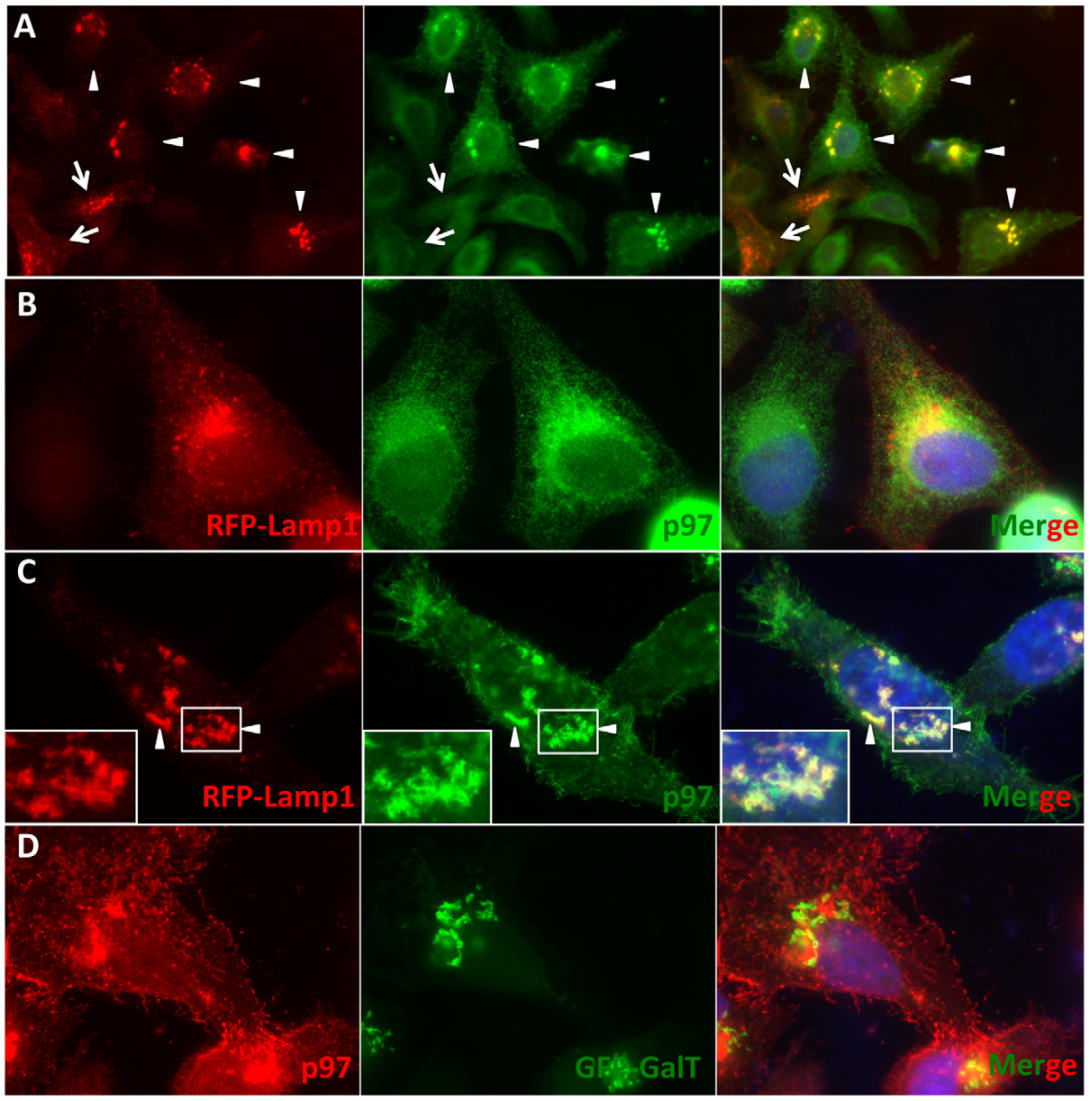
SVIP overexpression relocalizes lamp1 and p97/VCP to juxtanuclear vacuoles. A. SVIP induces RFP-lamp1 and p97/VCP colocalization. SVIP-overexpressing cells were identified by the characteristic relocalization of p97/VCP. Arrowheads: SVIP and RFP-lamp1 plasmids-transfected cells. Arrows: cells transfected with RFP-lamp1 plasmid alone. B, C. High magnification of cells expressing RFP-lamp1 alone (B) or RFP-lamp1 and SVIP (C). Arrowheads and insets: colocalization of p97/VCP and RFP-lamp1 in juxtanuclear vacuoles. Endogenous p97/VCP was labeled using monoclonal anti-p97/VCP antibody. D. The p97/VCP vacuoles do not colocalize with trans Golgi network labeled by GFP-GalT. Nuclei were counterstained in blue using DAPI.

Previous studies demonstrated that SVIP overexpression induces vacuolation of cells and that the vacuoles seem to be derived from the endoplasmic reticulum (ER) [5]. Interestingly, these changes are reminiscent of the relocalization of p97/VCP to the juxtanuclear vacuoles seen in SVIP overexpression cells (Fig. 2A, arrow). To determine the identity of p97/VCP-positive vacuoles, we cotransfected plasmids expressing SVIP and RFP-lamp1 (as a marker for lysosomes) in HeLa cells. SVIP-overexpressing cells were identified by the characteristic relocalization of p97/VCP in cells as shown in Figure 2A and B. The results showed that when expressed alone, RFP-lamp1 was localized in vesicles throughout the cells with more in the perinuclear region (Fig. 3A, arrows and 3B). Overexpression of SVIP induced accumulation of RFP-lamp1 to juxtanuclear regions (Fig. 3A, arrowheads in left panel and 3C) where it colocalized with the p97/VCP-positive vacuoles (Fig. 3A and 3C, arrowheads). To exclude the possibility that the perinuclear p97/VCP is localized to the Golgi, we cotransfected cells with plasmids encoding GFP-galactosyltransferase (GalT) as a Golgi marker. We found that the SVIP-induced p97/VCP-positive vacuoles were distinct from the Golgi network (Fig. 3D). These results indicate that the p97/VCP-positive vacuoles are localized to lysosomes.

### SVIP regulates autophagy

**Figure 4:**
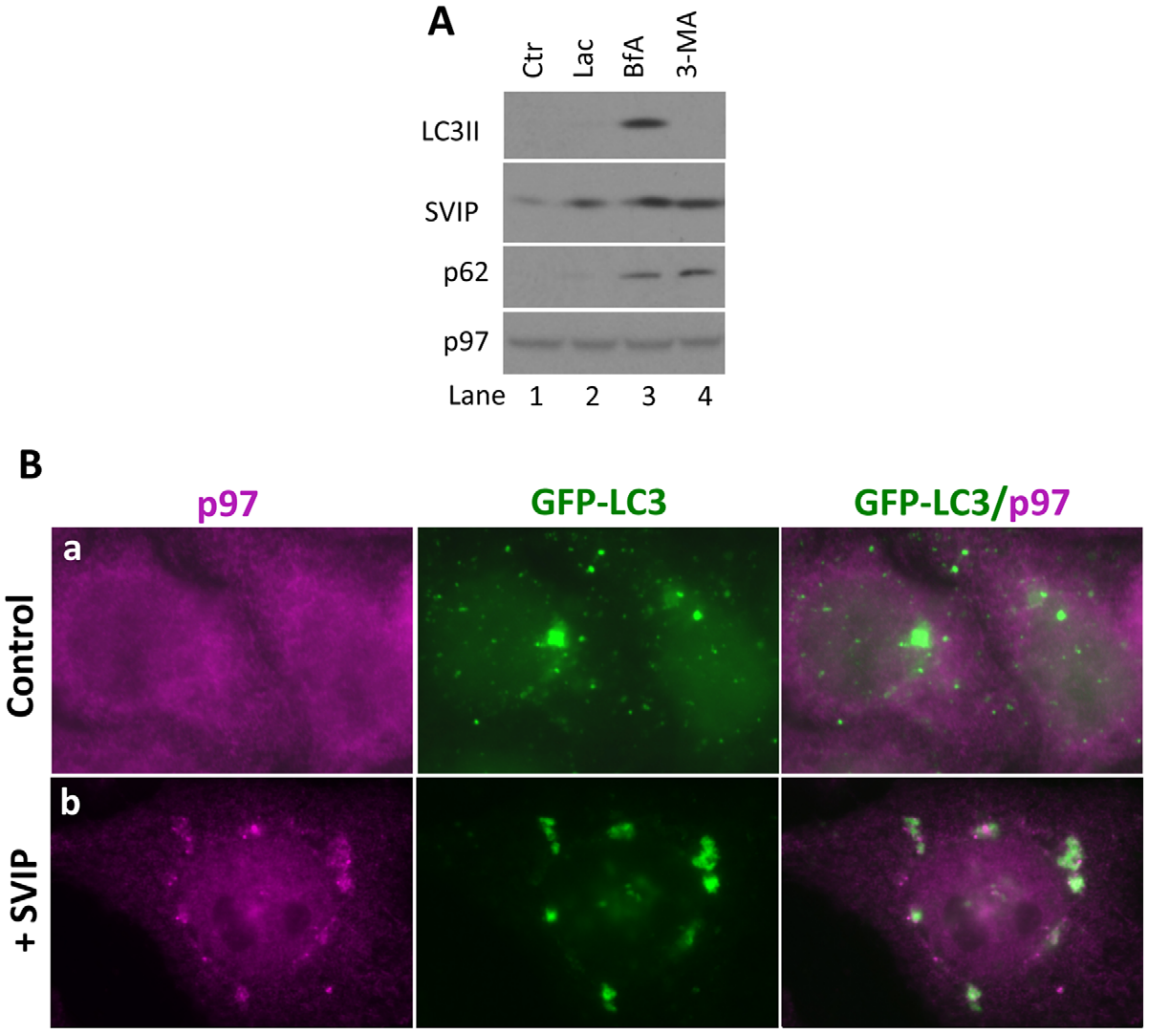
SVIP is degraded in part by autophagy and promotes colocalization of p97/VCP with GFP-LC3. A. Endogenous SVIP is degraded by both autophagy and the proteasomal pathways. HeLa cells were treated with BfA or 3-MA to inhibit autophagy or lactacystin to inhibit proteasome for 2 hours, and then processed for immunoblotting for indicated proteins. B. SVIP overexpression induces colocalization of GFP-LC3 with p97/VCP. GFP-LC3-expressing HeLa cells transfected with plasmid encoding SVIP or empty vector as a negative control were stained for endogenous p97/VCP.

Because lysosomes fuse with autophagosomes during macroautophagy we examined whether SVIP regulates autophagy. To test this possibility, we first asked whether SVIP itself is degraded by the lysosomes through autophagy pathway. We treated HeLa cells with two autophagy inhibitors: 3-methyladenine (3-MA) and bafilomycin A1 (BfA) [20], [21]. 3-MA acts at the early step of autophagy by blocking autophagosome formation and LC3 lipidation through inhibition of the type III phosphatidylinositol 3-kinases (PI3K). 3-MA treatment decreased LC3II and accumulated SVIP (Fig. 4A, lane 4 vs. 1), indicating that autophagosome formation is required for SVIP degradation. Therefore, SVIP is degraded by autophagy. BfA specifically inhibits vacuolar type H+-ATPase (V-ATPase) in cells, thereby inhibiting lysosomal degradation by hampering acidification of lysosomes. BfA treatment accumulated both SVIP and LC3II (Fig. 4A, lanes 3 vs. 1), which support the involvement of autophagy pathway in SVIP degradation. As a positive control, p62, which is a regulator of autophagy and also an autophagy substrate, was stabilized by treatment of cells with either 3-MA or BfA (Fig. 4A, lanes 3 & 4 vs. 1). We also assessed the involvement of proteasomes in SVIP degradation. Treatment of cells with the proteasome inhibitor, lactacystin, also accumulated SVIP protein (Fig. 4A, lane 2 vs. 1), indicating that the proteasome is also involved in SVIP degradation. It is worth noting that only LC3II was detectable in HeLa cells by immunoblotting under our experimental conditions. To further assess the involvement of SVIP in autophagy, we studied the effects of SVIP overexpression on GFP-LC3 localization in cells. HeLa cells stably expressing GFP-LC3 were transfected with plasmid encoding SVIP or empty vector as a negative control. The cells transfected with the empty vector contained GFP-LC3 puncta that were mostly small in size and dispersed throughout the cytoplasm and did not colocalize with p97/VCP (Fig. 4B, a). By contrast, cells transfected with the SVIP cDNA contained larger GFP-LC3 positive puncta in juxtanuclear regions of the cell where they colocalized, though not identically, with p97/VCP-positive vacuoles and vesicles (Fig. 4B, b). These results suggest that SVIP may be a regulator of autophagy.

### SVIP increases LC3 lipidation and autophagy

**Figure 5:**
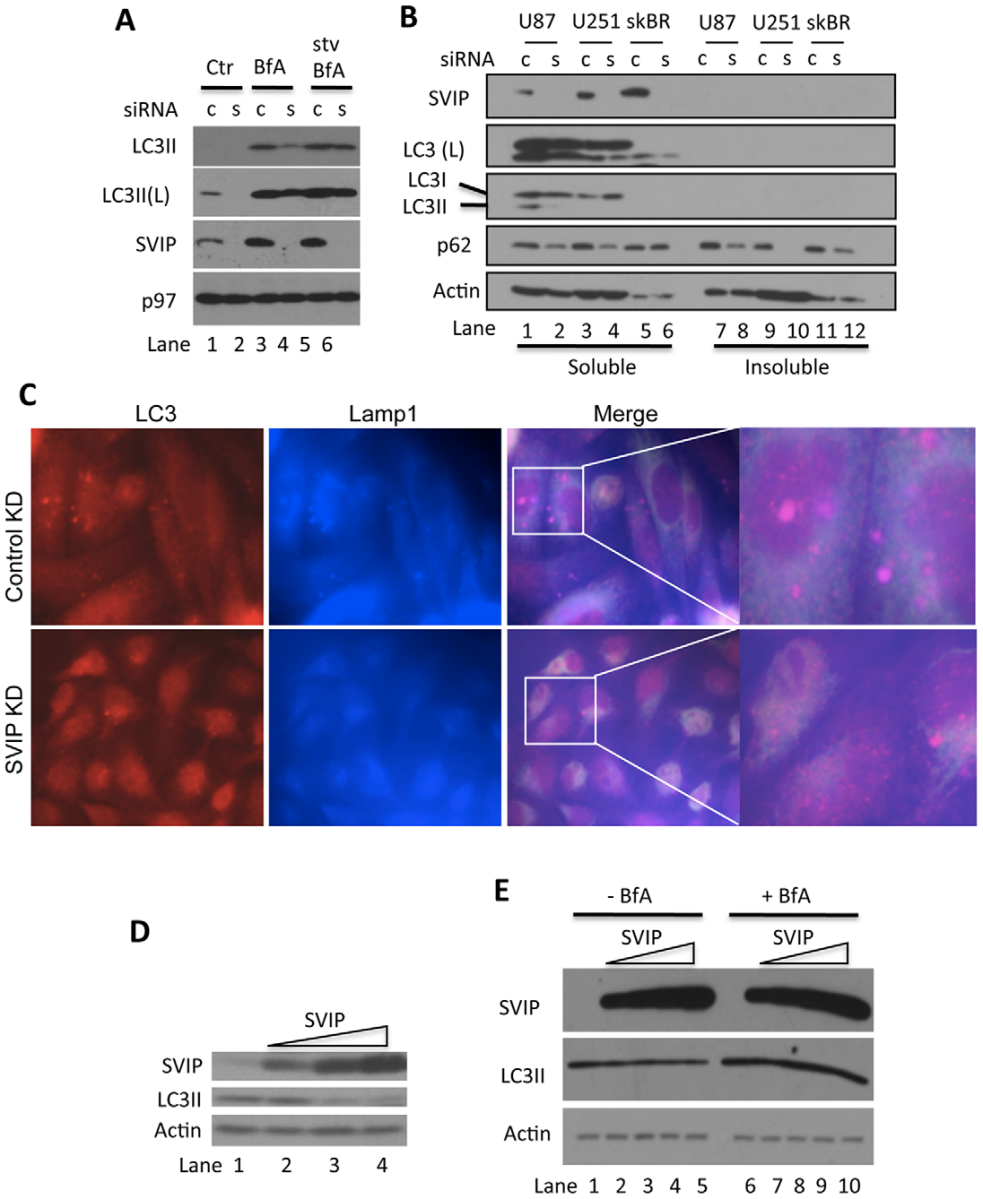
SVIP regulates LC3 lipidation. A. Knockdown of SVIP reduces the levels of LC3 lipidation in HeLa cells. Cells with knockdown of SVIP were treated with BfA or BfA and starvation for 2 hours followed by processing for immunoblotting for indicated proteins. LC3II(L): longer exposed blot. B. Knockdown of SVIP reduces the levels of LC3 lipidation in U87, U251 and SKBR3 cells. Cells with SVIP knockdown were separated into Triton X-100 soluble and insoluble fraction (see Materials and Methods for details) followed by processing for immunoblotting for indicated proteins. c: control siRNA; s: SVIP siRNA. LC3(L): longer exposed blot. C. SVIP knockdown decreases the formation of LC3-positive autophagosomes and reduces the aggregation of lysosomes. HeLa cells with control or SVIP knockdown were processed for immunofluorescent staining for LC3 (red) and lamp1 (blue). D. Overexpression of SVIP decreases LC3II levels. HeLa cells transfected with increasing amounts of SVIP plasmid were processed for immunoblotting as indicated. E. BfA accumulates more LC3II in SVIP-overexpressing cells. HeLa cells transfected with increasing amounts of SVIP plasmid were treated with BfA for 2 hours. Treated and untreated control cells were processed for immunoblotting for indicated proteins. c: control siRNA; s: SVIP siRNA.

Next, we determined whether SVIP regulates LC3 lipidation. The expression of endogenous SVIP was knocked down in HeLa cells by RNA interference (RNAi). The siRNA used to target SVIP mRNA has been reported previously [6]. SVIP knockdown significantly reduced the levels of LC3II (Fig. 5A, 2^nd^ panel, lane 2). To determine whether the decrease was due to increased LC3II degradation, we treated control and SVIP knockdown cells with BfA to inhibit autophagy. As expected, BfA treatment increased the levels of LC3II in the control cells, but the increase was less pronounced in the SVIP knockdown cells (Fig. 5A, lane 4 vs. 3). SVIP knockdown also reduced the levels of starvation-induced LC3II accumulation in BfA-treated cells (Fig. 5A, lane 6 vs. 5). Thus, LC3II reduction in SVIP knockdown cells may reflect a decrease in LC3 lipidation or expression. To clarify this issue, we studied the effects of SVIP knockdown on LC3II accumulation in three more cell lines, including U87 and U251, two glioma cell lines, and SKBR3, a breast cancer cell line. To exclude the possibility that the decrease in LC3II levels was due to changes in detergent solubility of LC3II, Triton X-100 soluble and insoluble fractions were examined for the presence of LC3II protein. Immunoblotting revealed that SVIP knockdown decreased LC3II levels in all three cell lines and, as expected, all of the LC3 was present only in the Triton X-100 soluble fractions (Fig. 5B). Moreover, in glioma cells, the decreases in LC3II levels were accompanied by an increase in LC3I levels (Fig. 5B). These results suggest that knockdown of SVIP does not change the total levels of LC3 rather reduces LC3 lipidation. The reduction in LC3II levels in SVIP knockdown cells correlates well with decreases in the formation of LC3-positive autophagosomes as revealed by immunofluorescent staining (Fig. 5C). Aggregation of lysosomes as revealed by staining for lamp1 was also decreased in SVIP knockdown cells (Fig. 5C). Interestingly we have shown earlier that SVIP overexpression promoted lysosomal aggregation (Fig. 3). Next, we ask if SVIP overexpression has an effect on LC3 lipidation. Increasing amounts of plasmids encoding SVIP were transfected into HeLa cells and then the cells were processed for immunoblotting for LC3. SVIP overexpression decreased the levels of LC3II in a dose-dependent fashion (Fig. 5D, E). When autophagy was inhibited with BfA, LC3II accumulated more than that in cells that did not overexpress SVIP (Fig. 5E, lanes 7–10 vs. 2–5). These results suggest that SVIP overexpression decreases LC3II accumulation due to increased autophagic degradation.

### SVIP regulates the levels of p62 expression

**Figure 6:**
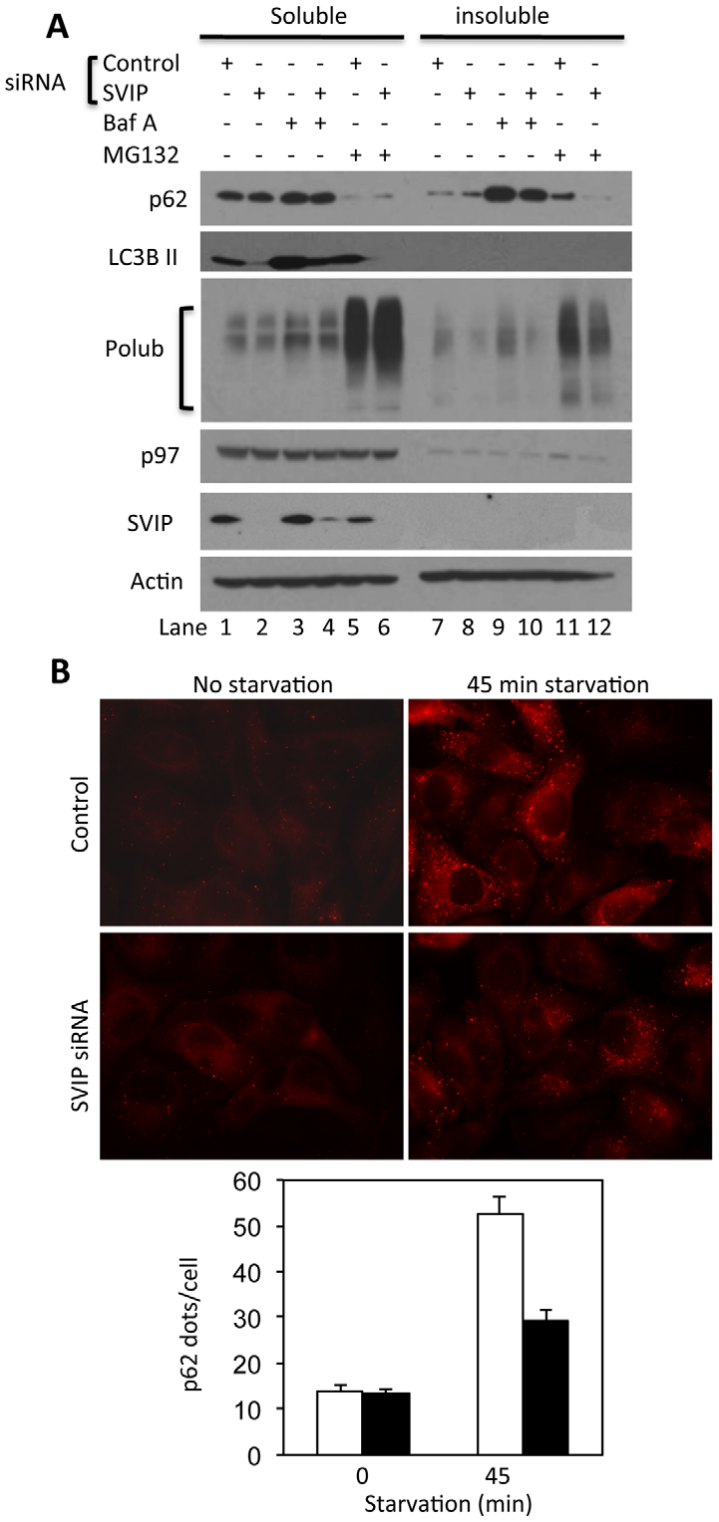
SVIP knockdown reduces the levels of p62 protein and decreases starvation-induced formation of p62 bodies. A. SVIP knockdown reduces the levels of p62 and polyubiquitinated proteins in detergent-insoluble fractions. Control and SVIP knockdown HeLa cells treated with BfA or MG132 were fractionated into Triton X-100 soluble and insoluble fractions followed immunoblotting for indicated proteins. B. SVIP knockdown decreases starvation-induced formation of p62 bodies. Upper panel: immunofluorescent staining of p62 bodies, lower panel: Quantification of p62 bodies per cell. p62 bodies were counted in 15–30 cells per field and repeated in 5 different fields. Error bars represent standard division calculated for 5 fields.

p62 sequesters ubiquitinated proteins in autophagosomes by simultaneous binding of ubiquitin-conjugates tagged onto proteins and LC3II proteins [17]. The autophagosomes eventually fuse with lysosomes resulting in degradation of ubiquitinated substrates along with p62 itself [17]. Therefore, degradation of p62 is a widely used as an indicator of autophagic activity. The decreased lipidation of LC3 in SVIP knockdown cells led us to predict a buildup of p62 protein. Surprisingly, knockdown of SVIP led to a decrease in total p62 protein levels in all three cell lines examined (Fig. 5B). To determine how SVIP knockdown causes p62 decrease, we first conducted RT-PCR to analyze p62 gene transcription in control and SVIP knockdown cells. The results showed that SVIP knockdown decreased the levels of p62 mRNA (Fig. S1), suggesting that reduced p62 transcription contributes to the decrease in p62 protein levels. Next, we assessed whether increased turnover contributes to the decreases in p62 protein. We treated SVIP knockdown HeLa cells with BfA to block autophagy or MG132 to inhibit proteasome. Immunoblotting showed that although the steady-state levels of p62 protein were not changed in HeLa cells, treatments with these inhibitors revealed a reduction of p62 protein in detergent-insoluble fraction in SVIP knockdown cells (Fig 6A, lane 10 vs. 9, and lane 12 vs. 11), suggesting that the synthesis of p62 protein is decreased, probably due to reduction of p62 mRNA. The steady-state levels of p62 protein were decreased in U87, U251 and skBR cells following SVIP depletion (Fig. 5B) but no change in HeLa cells (Fig. 6A). We reason that this difference may be due to inhibition of autophagic degradation of p62 caused by SVIP knockdown in HeLa cells. If the rates of p62 degradation were the same in control and SVIP knockdown cells, we would see a decrease in p62 level in SVIP knockdown cells. However, SVIP knockdown also leads to inhibition of p62 degradation, which should increase the level of p62 protein. These two opposite effects of SVIP knockdown on p62 protein levels result in the similar steady-state levels of p62 protein seen in control and SVIP knockdown HeLa cells. This is supported by the fact that when autophagy is blocked, the total level of p62 protein is lower in SVIP knockdown cells than that in control cells (Fig. 6A, lanes 10 & 4 vs. 9 & 3). In addition, the amount of detergent-insoluble polyubiquitinated proteins was also decreased in SVIP knockdown cells, which could not be reversed by autophagy or proteasome inhibition (Fig. 6A). The more pronounced decrease in p62 protein in MG132-treated cells is likely due to proteasome inhibition-activated autophagy, since previous studies reported that inhibition of proteasomal degradation activates autophagy [22]. These results suggest that SVIP knockdown decreases p62 protein levels by reducing its gene transcription and that SVIP may cooperate with p62 in sequestration of polyubiquitinated substrates.

### SVIP enhances starvation-induced autophagy

**Figure 7:**
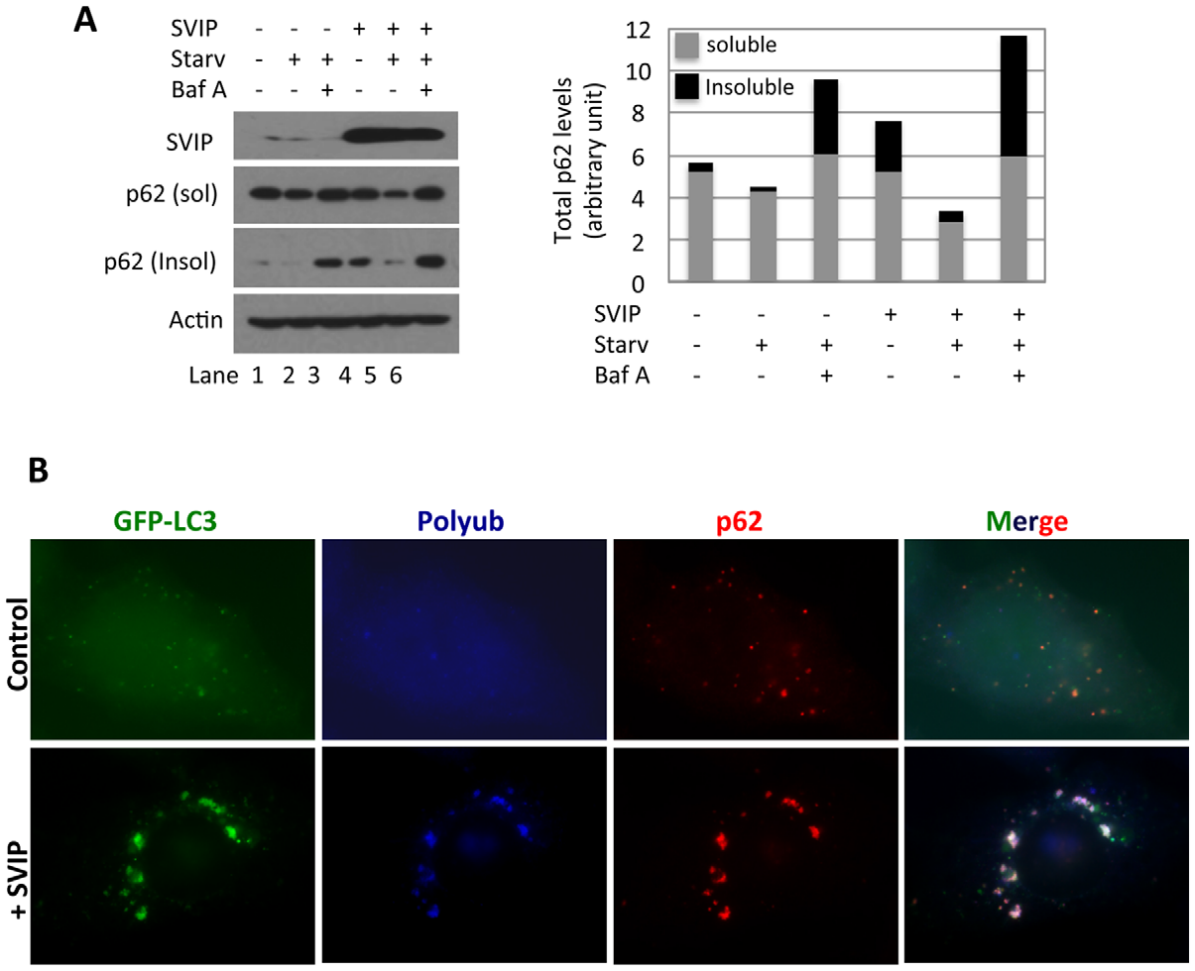
SVIP overexpression increases p62 protein levels and starvation-induced autophagy as well as promotes sequestration of p62 and polyubiquitinated proteins into autophagosomes. A. SVIP overexpression increases p62 in insoluble fraction and enhances starvation-induced autophagy. Control and SVIP-transfected HeLa cells were nutrient-starved in the presence or absence of BfA for 2 hours and then separated into Triton X-100 soluble (sol) and insoluble (insol) fractions followed by immunoblotting. Densitometry of p62 bands were expressed in the graph. B. SVIP overexpression promotes sequestration of p62 and polyubiquitinated proteins to autophagosomes. HeLa cells expressing GFP-LC3 transfected with plasmid encoding SVIP or empty vector were processed for immunofluorescent staining as indicated.

Decreases in p62 levels in SVIP knockdown cells prompted us to determine the changes in starvation-induced formation of p62 bodies. We starved HeLa cells in which SVIP was either targeted or not targeted for knockdown. Following the treatments the cells were fixed and examined for the number of p62 bodies by immunofluorescent staining and microscopy. SVIP knockdown resulted in no change in the basal level of p62 bodies, but we observed a significant decrease in starvation-induced formation of p62 bodies (Fig. 6B), which correlates with the reduction in the levels of p62 protein seen after SVIP knockdown HeLa cells (Fig. 6A), Next, we determined the effects of SVIP overexpression on starvation-induced autophagic degradation of p62. Control and SVIP overexpression cells were starved for 2 hours in the absence or presence of BfA. Cells were then lysed and separated into Triton X-100 soluble and insoluble fractions. Immunoblotting showed that SVIP overexpression increased the levels of total p62 protein and a further increase was observed in cells treated with BfA (Fig. 7A, lane 6 vs. 4 and 1, and graph). Interestingly, the increase was largely in the insoluble fraction (Fig. 7A, lanes 4, 6 and graph). This is in contrast to the effect of SVIP knockdown that caused decrease in p62 in insoluble fraction (Fig 6A, lanes 10, 12). When cells were subject to starvation, the levels of p62 protein decreased (Fig. 7A, lane 2 vs. 1 and 5 vs. 4). As expected, the starvation-induced decrease in p62 levels could be blocked by BfA treatment, consistent with p62 degradation by autophagy (Fig. 7A, lane 3 vs. 2 and 6 vs. 5). Importantly, compared with the control, SVIP overexpression increased p62 degradation, as evident by a larger decrease in total levels of p62 and a larger increase upon BfA-treatment (Fig. 7A, lane 5 vs. 2). Next, we determined the effect of SVIP overexpression on the formation of p62 bodies. Plasmid encoding SVIP was transfected into HeLa cells that stably express GFP-LC3. Transfected cells were subject to double immunofluorescent staining for polyubiquitin (blue) and p62 (red). GFP-LC3, polyubiquitin and p62 colocalized in cytoplasmic puncta in control cells (Fig. 7B). SVIP overexpression promoted colocalization of GFP-LC3, polyubiquitin and p62 to large perinuclear structures (Fig. 7B). We have shown earlier that these structures colocalize with lysosomes (Fig. 3C). Together, these results suggest that SVIP promotes p62-mediated targeting of polyubiquitinated proteins to the autophagy pathway.

## Discussion

In this study, we demonstrate a novel function of SVIP in regulation of p97/VCP subcellular localization and its regulatory role of autophagy. We found that SVIP overexpression markedly relocalizes p97/VCP from the cytosol to the plasma membrane, cytoplasmic foci and juxtanuclear vacuoles. SVIP overexpression also induces colocalization of GFP-LC3, p62, polyubiquitin and lamp1 to the p97/VCP-positive cytoplasmic foci and juxtanuclear vacuoles. Functionally, endogenous SVIP regulates autophagy by facilitating LC3 lipidation, enhancing p62 expression and sequestration of polyubiquitinated proteins to autophagosomes. SVIP promotes autophagy as evident by its ability to increase starvation-induced degradation of LC3II and p62 proteins.

We previously reported that SVIP is anchored to membranes through myristoylation and that it also interacts with p97/VCP via its p97/VCP-interacting motif (VIM) [6]. Relocalization of p97/VCP requires an intact VIM and the myristoylation site in SVIP, indicating that SVIP relocalizes p97/VCP to intracellular membrane structures through a direct interaction. However, although SVIP induces p97/VCP relocalization, it does not always colocalize with p97/VCP. For example, the p97/VCP-positive juxtanuclear vacuoles and the tips of the spine-like structures on the plasma membrane do not have SVIP (Fig. 2A, B). We speculate that once SVIP brings p97/VCP to membrane, it may dissociate from p97/VCP and p97/VCP may in turn bind to membrane through another protein. The similar regulation may occur in neurons. Immunofluorescent staining of rat spinal cord sections demonstrated that p97/VCP is enriched at the cell periphery, presumably at the plasma membrane of neurons, whereas SVIP is largely in the cytoplasm (Fig. 1B, insets). SVIP is highly expressed in central nervous system, suggesting that SVIP-mediated p97/VCP localization may be important for neuronal function. How this regulation occurs and its functional importance remain to be determined. Although SVIP induces p97/VCP localization to lysosomes, it was not degraded in SVIP-overexpressing cells, suggesting that it coats the cytosolic surface of the vesicles (foci). Moreover, some p97/VCP-positive vesicles were readily seen in close contact with lysosomes (Fig. 3C) and GFP-LC3-positive structures (Fig. 4B, b). We speculate that the p97/VCP-positive vesicles may play a role in delivery of their contents to lysosomes for degradation.

We obtained evidence suggesting that SVIP regulates basal autophagy. Knockdown of SVIP decreases LC3 lipidation and reduces p62 mRNA and protein levels. The changes in LC3 and p62 in SVIP knockdown cells decreases starvation-induced autophagy activity as reflected by reduced formation of p62 bodies. Conversely, overexpression of SVIP increased LC3 lipidation and increased p62 protein levels as well as enhanced sequestration of polyubiquitinated proteins and p62 into autophagosomes. These increases correlate well with enhancement of starvation-induced p62 degradation. Interestingly, we found that the increases in p62 protein in SVIP-overexpressing cells and the decreases in p62 proteins in SVIP-knockdown cells occurs largely in detergent-insoluble fraction, suggesting that the enhanced sequestration of p62 and polyubiquitinated proteins in autophagosomes may also increase their aggregation. In support, we found that knockdown of SVIP decreases p62 protein and polyubiquitinated proteins largely in insoluble fraction. Overexpression of SVIP promotes colocalization of p62, polyubiquitinated proteins, GFP-LC3 and lamp1 to the juxtanuclear region. These colocalizations may facilitate the fusion of autophagosomes with lysosomes. This is consistent with the recent studies showing that perinuclear positioning of lysosomes enhances autophagy by influencing and coordinating mTOR activity, autophagosome biogenesis and autophagosome-lysosome fusion [23]. Thus, SVIP may be one of the regulators of lysosome perinuclear positioning. In addition, ER stress has been shown to activate autophagy [24], [25]. We reported previously that SVIP overexpression inhibits ERAD, thereby inducing ER stress [6]. Thus, SVIP overexpression may also activate autophagy via inducing ER stress. Interestingly, we previously found that prolonged ER stress upregulates endogenous SVIP accompanied by an increase in ER stress [6]. It is reasonable to speculate that the SVIP upregulation may activate autophagy to enhance degradation of misfolded proteins caused by prolonged ER stress.

## Materials and Methods

### Antibodies, siRNAs, and plasmids

The following primary antibodies were used: monoclonal anti-p62 (Cell Signaling), rabbit anti-LC3B (Cell Signaling), mouse monoclonal anti-p97/VCP (Affinity Bioreagents), anti-β-actin (Sigma), anti-ubiquitin FK2 (Millipore), anti-Lamp1 (Developmental Studies Hybridoma Bank), polyclonal anti-p62 (Sigma), and polyclonal anti-SVIP. Secondary antibodies conjugated with Alexa Fluor 350 (blue), Alexa Fluor 488 (green) and Alexa Fluor 594 (red) were purchased from Invitrogen. Control siRNA and siRNAs targeting human p97/VCP and SVIP have been previously reported [6]. Plasmids for expressing wt SVIP and its G2A and VIM mutant have been previously described [6]. pEGFP-GalT plasmid was a gift from Dr. Yanzhuang Wang. RFP-lamp1 plasmid was acquired from Addgene.

### Cell culture and transfection

HeLa [26], U251 and U87 [27], and HEK293 [28] cells as well as HeLa cells stably expressing GFP-LC3 [29] were cultured in DMEM (Dulbecco's modified Eagle's medium) supplemented with 10%(V/V) fetal bovine serum, 50 units/ml penicillin, 50 µg/ml streptomycin and Glutamine under 5% CO2 in a humidified incubator. SKBR3 cells were cultured in DMEM/F12, with 10% FBS and the same supplements. Transient transfection was done using calcium-phosphate precipitation and Lipofectamine 2000 (Invitrogen). For RNA interference (RNAi), siRNA was transfected using Lipofectamine 2000. MG132, 3-MA and BfA were added to the culture medium for the indicated time at a final concentration of 10 µM, 2,5 mM and 100 ng/ml, respectively. Nutrient-starvation was done by washing cells with PBS X3 times followed by culturing the cells in Hank's Balanced Salt Solution for 2 hours.

### Immunofluorescence

HeLa cells or cryostat sections (7 µm thick) of mouse spinal cord were fixed with 4% paraformaldehyde in 1X PBS at 4°C for 30 minutes, and then subject to immunofluorescent staining as previously reported [28].

### Immunoblotting

To extract total proteins, cells or homogenized mouse organs were lysed in SDS lysis buffer (50 mM Tris/HCl pH 8.0, 150 mM NaCl, 1 mM EDTA, 1% NP-40, 1% SDS). To separate cell lysate into Triton X-100 soluble and insoluble fractions, cells were lysed in Triton X-100 lysis buffer (50 mM Tris/HCl pH 8.0, 150 mM NaCl, 1 mM EDTA, 1% NP-40, 1% Triton X-100) on ice for 30 minutes, then centrifuged at 14,000 rpm for 10 minutes. The resulting pellet was lysed in the SDS lysis buffer as insoluble fraction. The supernatant was used as soluble fraction. Immunoblotting was done as previously reported [28].

### RT-PCR

Total RNA was extracted from control and SVIP knockdown SKBR3 cells using TRIzol reagent following the manufacturer's instruction. RT-PCR was done as reported previously [30]. The primers set used for p62 were 5′- CGTCCTCCTCGTGCAGGGGA-3′ (forward) and 5′-GGTCAGCCTCTGGCGGGAGA-3′ (reverse). We performed 25 and 30 cycles of PCR to reduce the effect of amplification saturation.

## Supporting Information

Figure S1 **SVIP knockdown decreases the mRNA levels of p62 in SKBR3 cells.** p62 mRNA was amplified by RT-PCR for 25 and 30 cycles.
(TIF)
