# Supplementary figures and images for: SVIP Induces Localization of p97/VCP to the Plasma and Lysosomal Membranes and Regulates Autophagy
Source: PLoS One. 2011 Aug 31;6(8):e24478. doi: 10.1371/journal.pone.0024478 (PMC3164199; doi:10.1371/journal.pone.0024478)

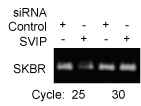

Supplement: Figure S1 — SVIP knockdown decreases the mRNA levels of p62 in SKBR3 cells. p62 mRNA was amplified by RT-PCR for 25 and 30 cycles. (TIF) [file pone.0024478.s001.tif]
